# Why Reducing Low-Value Care Fails to Bend the Cost Curve, and Why We Should Do it Anyway

**DOI:** 10.34172/ijhpm.2023.7803

**Published:** 2023-05-27

**Authors:** Daniëlle Kroon, Niek W. Stadhouders, Simone A. van Dulmen, Rudolf B. Kool, Patrick P.T. Jeurissen

**Affiliations:** Radboud University Medical Center, Radboud Institute for Health Sciences, IQ healthcare, Nijmegen, The Netherlands

## Background

 Low-value care provides either a modest benefit or even no benefit at all to the patient, while it is potentially harmful, may not reflect patient preferences, and may result in considerable costs. Several US studies have estimated the nationwide costs of medical waste, including low-value care, at hundreds of billions of dollars.^1,2^ Health systems can reduce low-value care by targeted de-implementation interventions, of which many have been proven successful.^3,4^ Studies into de-implementation regularly estimate substantial cost savings to the health system, claiming it will save society millions of dollars. For example: a de-implementation strategy reducing vitamin D testing could directly save up to 1.5 million Canadian dollars per year in Alberta^5^; four Dutch departments of internal medicine could save 1.2 million euros a year by reducing inappropriate laboratory testing^6^; avoiding inappropriate imaging could save Massachusetts 50-100 million dollars annually^7^; and stopping five low-value general surgery services could save the English National Health Service (NHS) over 150 million euros per year.^8^ Evidently, these potential savings attracted the attention of policy-makers. Reducing low-value care has been adopted, for example, by the Dutch government to ‘bend the healthcare cost curve’ while simultaneously increasing the quality of care. Could this be the panacea to ailing health systems? Or are these promises too good to be true? In this perspective, we argue that calculating generous savings by reducing low-value care is wishful thinking.

 It is frequently assumed that de-implementing low-value care practices causes a decline in the total volume of the provided care, which induces cost savings. These savings are commonly estimated based on the average costs of a care practice, and subsequently interpreted as direct societal cost savings. This reasoning suffers from several fallacies. We address four mechanisms that provide insight into why the actual savings potential is substantially lower than these standard calculations.

###  1. Care Substitution

 Roemer’s law — a bed built is a bed filled — states that hospital utilization depends on the availability of care.^9^ De-implementation of low-value care can induce care substitution. For example, de-implementation of knee arthroscopies for patients with degenerative osteoarthritis frees up the time of an orthopedic surgeon, other team members, and the operating theatre. This opens up capacity for other surgical procedures. If these are of high value, it results in more overall value for approximately the same price. But de-implementation can also provoke a supplier-induced demand of other low-value services: the orthopedic surgeon may perform more low-value shoulder operations.^10^ The gains of de-implementation evaporate in this scenario. In order to reduce the spending, the essential first step is to reduce the volume of care.^11^ Thus, care substitution should be discouraged. In the real world of the internal budgetary politics in hospitals, however, active volume reductions are rare.^12^ Healthcare organizations need to decide and actively plan how the freed capacity will be used, otherwise de-implementation will neither increase value-for-money nor result in cost savings.

###  2. Not All Estimated Savings Are Realistic

 The estimated savings of de-implementation interventions are frequently based on the list price or average unit cost of a care practice.^2,6-8^ These estimations are, however, not representative of potential savings over either the short term or the long term. This can be explained by dividing hospital costs into three layers^13^:

1. *Variable costs* are the costs of disposable equipment, drugs and medical devices that can be reduced fully and immediately upon de-implementation. This category represents the direct cost savings of reducing low-value treatments.
2. *Semi-variable costs* are all costs that can be lowered if a sufficient reduction in the number of care practices is realized, for example, salary costs of hourly employees. In such cases, a threshold of low-value procedures needs to be met: the minimal number of reduced procedures before one can reduce work hours and subsequently also costs. For some costs, it takes a substantial time span to reach this threshold. For example, for the purchasing costs of reusable medical devices and the salary costs of permanent employees. However, in the long term all semi-variable costs have the potential to be reduced.
3. *Fixed costs* are in essence insensitive to volume changes. For example, building-related costs and expenses on organizational overhead, like administration and information and communications technology. Such costs can be reduced by taking certain measures, but in this definition the opportunities for downsizing do not depend on the care volume.

List prices or average unit costs of care practices cover all three cost categories, but the expected savings on the short term are only a small part of this amount. Roberts et al estimated that the true variable costs account for only 16% of all hospital expenditures.^14^ The variable costs are also relatively low for non-hospital care, such as diagnostic tests and general practitioner consultations. The remaining non-variable costs do not ‘disappear’ automatically: time is required before they can be reduced along with specific strategies that can be quite unpopular such as reducing their professional autonomy or scaling down workforce.^15^ And despite all efforts, fixed costs remain since these are independent of volume changes.

 In addition, reaching the threshold to reduce semi-variable costs is challenging. First, it could be difficult to determine relevant thresholds. With planned care such as cataract surgeries, hospitals could schedule fewer procedures and eventually meet the threshold to realize cost savings. This is, however, not an option for emergency or semi-acute care, such as trauma surgery. Hospitals need to have a minimum workforce to be able to cover peaks in this type of unplanned care. For some staff, particularly specialized doctors and nurses in small-hospital settings, scaling down is often not a viable option (ie, those staff are part of hospital fixed costs).

 Secondly, organizational resistance can prevent reaching the threshold to reduce staff. Waiting lists are a defense against cost-cutting management. Scaling down staff and capacity, while there is sufficient demand for care, can trigger resistance among professionals and patients: money is chosen over (valuable) care. In addition, long de-implementation periods hinder reaching thresholds. If ‘time’ becomes available, professionals will take up other tasks since doing nothing may undermine their professional integrity.^16^ Their perceived workload will therefore not be reduced when the theoretical threshold is reached, also causing organizational resistance against scaling down workforce.

###  3. Payment Systems Hinder Wide-Scale De-implementation

 The vast majority of the healthcare systems partly rely on fee-for-service elements to incentivize adequate volume and prevent waiting lists. Fee-for-service systems contain a major financial disincentive for de-implementation. Especially on the short and medium term the loss of revenue exceeds the amount of cost savings. Healthcare organizations have to either increase the prices of other care or substitute de-implemented care to avoid financial distress after large-scale de-implementation. Either way, the societal cost savings will be lower than the saved reimbursements because of such compensation methods.

 This especially applies for payment systems in which the total hospital budget entirely depends on the volume. However, even global payment systems such as the NHS in the United Kingdom rely on fee-for-service elements, for example when they contract private providers or when they seek to reduce patient backlogs. In payment systems with fixed hospital budgets, de-implementation typically does affect hospital income in a less severe way, and any cost savings might increase profit margins. However, also in these cases a decrease in care volume does not automatically result in societal savings. To achieve societal savings, the hospital budgets should be reduced in response to de-implementation efforts. Shared-savings agreements are designed to do this and the results so far are promising.^17,18^ However, adjusting payment structures requires costly, complex and politically sensitive adjustments.

###  4. Reluctance of Funding De-implementation Costs 

 The success of de-implementation depends on a tailored strategy that requires (substantial) financial resources both upfront and during the process. Since there is also no guarantee that a healthcare organizations will succeed in reducing costs, the question is who is willing to invest in de-implementation. Hospitals and healthcare professionals are unlikely to take the lead if they have to invest and take on the financial risk, especially if they do not profit from any cost savings when revenues decline. In order to provide guarantees, multi-year fixed revenue contracts could be employed. However, such agreements risk ratchet-effects, where payers aim to capture full benefits after the agreement period. The government and healthcare insurance companies may want to invest, but only if real cost savings are rendered. Given all uncertainties, payers may also be unwilling to fund upfront investments in de-implementation. Furthermore, in multiple-payer models competitors may refuse co-funding, as they may free ride on other payers’ investments.

 In addition to the costs of the de-implementation strategy, the alternative for low-value care practices also requires funding. For example, instead of the chronic use of opioids for knee osteoarthritis, patients are advised to exercise under supervision of a physical therapist. The cost of the alternative care may be even higher than the cost of the low-value care. These expenses will reduce the potential societal payoffs of de-implementation.

 In all cases, one needs to take the de-implementation costs and costs of complementary care into account, otherwise the cost-saving effects will be too optimistic from a societal perspective. Moreover, the government needs to take responsibility and invest in de-implementation. Without its support, other payers and healthcare organizations are unlikely to join a major investment in large-scale de-implementation.

###  De-implementation For Sustainable Healthcare

 The sobering conclusion is that the savings potential of de-implementation interventions is unsure, but certainly considerably lower than claimed by policymakers and in the scholarly literature.^1,2,5-8,19^ Healthcare organizations face reimbursement reductions that will far exceed cost savings and require extensive efforts to realize. To overcome this, financial incentives of all stakeholders must be aligned, but this requires innovative payment methods and complex healthcare system changes.

 This does not mean we should stop de-implementing low-value care. While obtaining cost savings is challenging, it may be possible with a long-term business plan containing active planning to suppress substitution and to reduce semi-variable costs. Moreover, individual patients benefit from de-implementation through fewer adverse events and more high-value care. And from a societal perspective, de-implementation has the potential to increase the value of care and stimulate efficient use of time and resources in healthcare. It enables a capacity shift to high-value care. This shift will be more feasible to achieve than downscaling: actively substituting low-value care for high-value care faces less professional resistance than directly aiming for cost-savings. It does not result in substantial loss of revenue for providers, nor does it require a challenging reduction of semi-variable costs. In this scenario, the society benefits by more value for money regarding healthcare taxes and premiums. In addition, this shift is essential in light of increasing shortages of healthcare professionals in almost all countries.^20^ During the next decade, tough choices have to be made.^21^ If these choices are not made, healthcare quality and safety will be compromised, hurting vulnerable populations the most. Efficient use of healthcare resources is an important requirement for a sustainable health system.

 De-implementation of low-value care should not be adopted because of the opportunity for direct cost savings, but it should be enthusiastically embraced to improve the quality of care, reduce harm for patients, free up capacity for high-value procedures and to ensure future workforce sustainability.

## Ethical issues

 Not applicable.

## Competing interests

 Authors declare that they have no competing interests.

## Funding

 ZonMW, the Dutch Organization for Health Research and Development (grant number 80-83920-98-803) and the Dutch Ministry of Health, Welfare and Sport (grant number 331032).
